# Behavioral Observation Procedures and Tests to Characterize the Suitability of Sows for Loose-Housed Farrowing Systems

**DOI:** 10.3390/ani11092547

**Published:** 2021-08-30

**Authors:** Julia Neu, Nina Göres, Jelena Kecman, Barbara Voß, Frank Rosner, Hermann H. Swalve, Nicole Kemper

**Affiliations:** 1 Institute for Animal Hygiene, Animal Welfare and Animal Behavior, University of Veterinary Medicine Hannover, Foundation, 30173 Hannover, Germany; julia.neu@tiho-hannover.de (J.N.); nina.katharina.goeres@tiho-hannover.de (N.G.); 2 Institute of Agricultural and Nutritional Sciences, Martin Luther University Halle-Wittenberg, Theodor-Lieser-Str. 11, 06120 Halle, Germany; jelena.kecman@landw.uni-halle.de (J.K.); frank.rosner@landw.uni-halle.de (F.R.); hermann.swalve@landw.uni-halle.de (H.H.S.); 3 BHZP GmbH, An der Wassermühle 8, 21368 Dahlenburg, Germany; voss@bhzp.de

**Keywords:** aggression, behavior test, loose-housing, sow behavior, stockmanship

## Abstract

**Simple Summary:**

In this study, different behavior tests were developed and applied to characterize the behavior of sows against humans and piglets in systems with short-term fixation only. In loose-housed sows, it is of extreme importance that the sows neither attack the stockpersons nor crush their piglets through carelessness. Selecting the sows for the respective traits might show positive effects in a successful realization of these husbandry systems. For example, the Dummy Arm Test simulated catching the piglets. In the Towel Test, the general reaction to unknown stress situations was tested by throwing a towel towards the sow during a resting phase. Another test simulated the emptying of the trough to simulate interaction with humans during a routine procedure. The study showed that the majority of the sows reacted calmly. Nesting and lying behavior were also analyzed, as was the behavior of sows when their litters returned after a short separation. This study showed that the behavioral observation procedures and designed tests are suitable to characterize sows’ behavior towards humans and piglets with regard to traits that are particularly important in systems without fixation.

**Abstract:**

The objective of the study was to evaluate behavioral observation procedures and tests to characterize sows’ behavior for their suitability for free farrowing systems. Nest building activity (NB), lying-down behavior (LDB), and position after lying down (PLD) were assessed. Four tests were designed to characterize the reaction of sows to a novel object and an unexpected situation (Towel Test, TT), behavior towards humans (Dummy Arm Test, DAT; Trough Cleaning Test, TCT), and behavior towards piglets (Reunion Test, RT). The study was performed on a nucleus farm in 37 batches including 771 purebred landrace sows housed in farrowing pens with short-term fixation. The assessment of NB started 2 days before the expected date of the farrowing. In 56.2% of the observations, the sows showed increased chewing activity on gunnysacks. The LDB and PLD were assessed on days 3 and 19 post partum (p.p.). In 49.1% of the observations, sows showed careful lying-down behavior. In 50.1% of cases, sows preferred the stomach-teats-position when lying down. With the DAT on day 4 p.p., in 89.3% of observations, no or only slight reactions of the sow were documented. The TT and TCT were performed on days 3 and 10 p.p. Strong defensive reactions of animals towards humans were recorded in 4.5% of the observations in the TT, and in 4.0% of the observations in the TCT. In the RT on day 3 p.p., in 61.8%, a joyful response of the sows to the reunion with their piglets was observed. This study showed that the behavioral observation procedures and designed tests are suitable to characterize sows’ behavior towards humans and piglets with regard to traits that are particularly important in systems without fixation.

## 1. Introduction

The housing conditions of sows during farrowing and lactation are currently under discussion in some European countries, such as Germany, and systems without a permanent fixation of the sow must be implemented over the coming decades [1]. This not only presents the farmer with construction challenges but also takes the whole animal handling process to another level. Initially, farrowing crates were introduced to decrease piglet mortality due to crushing, simplify routine measures and management treatments by humans surrounding farrowing, and ensure work safety [2]. Due to these effects, individual housing and restriction of movement in farrowing crates for sows over the whole birth and lactation period is still common in most European countries. However, the restriction of the sows’ mobility over weeks has produced many serious concerns about animal welfare [3,4], which has led to legislative changes in Germany and other countries. Not only is the sow prevented from engaging in all other non-vertical movements, but her behavioral patterns, such as standing up, lying down, rooting, and nest building behavior, as well as the interaction between sow and piglets, are very limited in pens with crates. Permanent fixation leads to several impairments of the sows’ welfare [5] and restricts the expression of natural behavior [6]. On the other hand, the welfare of the piglets cannot be denied. Recent studies found increased piglet losses caused by piglet crushing in free farrowing pens compared to pens with farrowing crates even in the first three days after birth [7]. In contrast, other studies concluded that the total mortality of crushed piglets was comparable in free farrowing pens and pens with farrowing crates [8,9]. Systems with temporary confinement of the sow after birth represent a compromise for improving both the sow’s welfare and the piglets’ survival. One important question, however, is which character traits sows should possess to successfully be kept in these systems without permanent fixation or any fixation at all.

With reduced confinement, sows must respond to the signals of their piglets and react carefully to avoid crushing [10]. Moreover, sows’ behavior towards humans is essential when the sows are not fixated [11]. Maternal characteristics, also related to the expression of natural behavioral needs (e.g., nest building behavior), are important in terms of piglet mortality [12,13]. Furthermore, studies with various behavior tests showed the possibility of classifying maternal care. For instance, sows’ behavior was assessed after confrontation with various stimuli such as piglets’ screams caused by treatment [4,14,15,16], simulated piglets’ screams caused by crushing [12,14], and reaction to piglets’ screams and crushing of piglets during that time [17]. Moreover, sows’ reactions to separation from piglets and reunion were analyzed [12]. All of these studies showed individual variances. Other studies documented that sows perform many pre-lying behaviors to draw the piglets’ attention before lying down [18], as many losses are due to crushing after a transition from “standing to lying” [6]. However, the sow’s will to protect her piglets can represent a serious threat for humans entering the pens or handling the animals. If there is no possibility of fixating the sow for treatment or interaction, as is the case in many loose housing farrowing systems, the sow should be gentle, relaxed, and not aggressive. In addition, any aggressive behavior negatively affects animal welfare [15].

The aim of the present study was to develop, apply, and analyze the use of three behavioral observation procedures and four behavior tests to characterize the suitability of sows’ behavior for loose-housing pens, mainly towards humans, but also in other important traits concerning piglet survival and mothering abilities.

## 2. Materials and Methods

### 2.1. Animals and Housing

The present study was conducted on a nucleus farm (BHZP GmbH, Dahlenburg, Germany) managed in a 3-week batch farrowing system with a 4-week lactation period. All legal requirements for animal welfare on pig farms in the EU (Council Directive 2008/120/EC of 18 December 2008) and Germany (Tierschutzgesetz, Tierschutz-Nutztierhaltungs-Verordnung) were met. In compliance with European Directive 2010/63/EC Article 1 5. (f), the present study did not imply any invasive procedure or treatment of the animals. The study was reviewed and approved by the Animal Welfare Officer of the University of Veterinary Medicine Hannover, Foundation, Germany.

Data collection took place from October 2016 to December 2018. During 37 test batches, 771 purebred landrace sows with 1444 litters were involved in the study. The parity number of sows was 2.2 ± 1.3 (mean ± SD).

**Figure 1:**
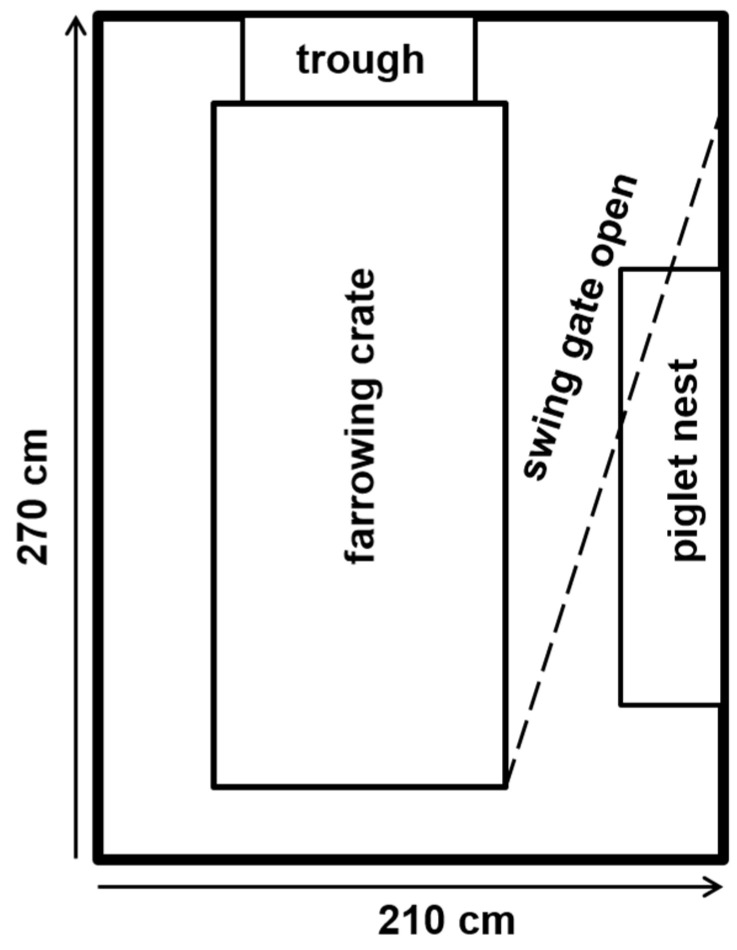
Schematic view of the free-movement pen with movable fixation (Big Dutchman International GmbH, Lower Saxony in Vechta, Germany).

One week before the expected farrowing date, sows were moved to the farrowing compartments. Each compartment included 40 single farrowing pens (Big Dutchman International GmbH, Lower Saxony in Vechta, Germany; Figure 1). The farrowing pen dimensions were 2.7 m × 2.1 m, which provided 5.67 m^2^ in total area. Each pen was equipped with a movable farrowing crate, a trough, and a nipple drinker for free access to water in the front. The sows were fixated in farrowing crates from 7 days ante partum (a.p.) until the 9th day (9.4 ± 1.6; test batches: 1–18) and 4th day (4.4 ± 1.0; test batches: 19–37) p.p., respectively. After that, and until the end of lactation, the farrowing crates were opened to provide the sow with additional space and freedom to move, including the ability to turn around. Depending on the lactation stage and parity, the sows received individual amounts of feed with an automatic feeding system (Spotmix, Schauer Agrotronic GmbH, Prambachkirchen, Austria).

The piglet nest with a heated resting area was located beside the farrowing crate, in the area which the sow could not access when the crate was closed. If necessary, heating lamps were hung over the piglet nests. Nipple drinkers for the piglets, with free access to water, were installed beside the piglet nest.

Litters were standardized at 14 piglets per sow by cross fostering where necessary within the first 24 h after birth. They were weighed individually and ear-tagged; their sex and any deviation from the norm were documented.

### 2.2. Behavioral Observation Procedures and Behavior Tests

Three behavioral observation procedures (NB, LDB, PLD) and four behavioral tests (TT, DAT, TCT, RT) were applied at different points in time during lactation to evaluate the sows’ reactions to various external stimuli (Table 1). All observations and tests were performed directly by two trained observers.

#### 2.2.1. Nest Building (NB)

The nest building activity of sows was investigated using a gunnysack hanging in the farrowing pen. These commercially available gunnysacks (burlap sacks, jute sacks; MS Schippers, Kerken, Germany) could be reached by the sow and had a size of approximately 110 × 60 cm. Two days before the expected farrowing, the gunnysacks were made available, and every morning until birth, the manipulation by the sow was evaluated, resulting in 3643 observations at three defined time points: 2 days a.p., 1 days a.p., and 0 days of farrowing. The sow’s NB activity was classified as being in one of 5 categories from “1” (no NB activity) to “5” (gunnysack was torn) (Table 2). Due to the very low occurrence of category 4 (0.5%) in the data set, categories 4 and 5 were combined into category 4.

#### 2.2.2. Lying-Down Behavior (LDB)

To determine any differences in the LDB of the sows, the manner of lying down was evaluated at two defined times: around day 3 p.p. (mean ± SD: 3.3 ± 1.0 days) in closed farrowing crates (1444 observations) and around day 19 p.p. (19.0 ± 1.3days) in open farrowing crates (1370 observations). This resulted in 2814 observations. The LDB of sows was classified from category ‘1’ (very cautious lying down) to category ‘4’ (careless lying down) (Table 2).

#### 2.2.3. Position after Lying Down (PLD)

The PLD was determined to analyze whether the sows had a favorite position (on the stomach, on the left or right body side) (Table 2) on which they laid down (1415 observations). The observation was carried out at the same time points as the LDB: around day 3 p.p. with closed farrowing crates (701 observations) and around day 19 days p.p. with open farrowing crates (714 observations).

#### 2.2.4. Towel Test (TT)

In the TT (2846 observations), the reaction of each sow to novel objects and unknown situations was determined by a towel suddenly thrown in the direction of the sow’s head during a resting period. The sow’s reaction was classified as being in one of three categories (Table 2). This test was performed around 3 days p.p. (3.4 ± 1.0 days) with closed crates and fixed sows (1444 observations) and around 10 days p.p. (9.8 ± 1.6days) with open loose-housed sows (1402 observations).

#### 2.2.5. Dummy Arm Test (DAT)

**Figure 2:**
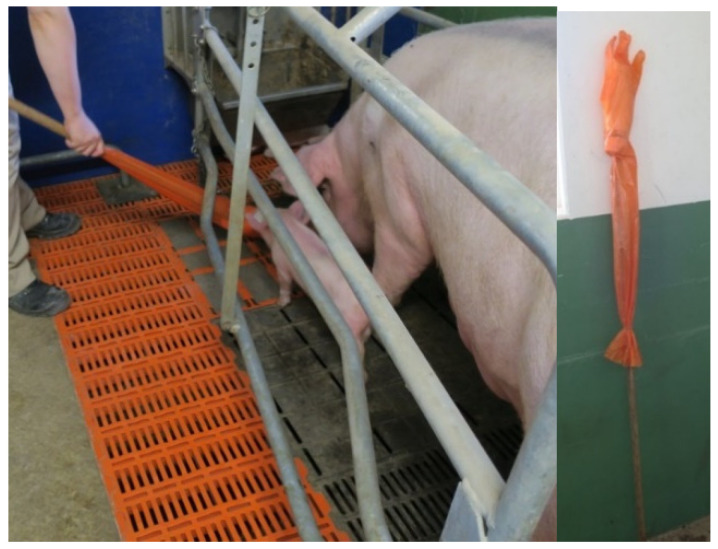
Performance of the Dummy Arm Test (DAT) (Photo: BHZP GmbH).

The DAT (1444 observations) was performed on 4 days p.p. (3.9 ± 1.0 d), while the sows were still fixed in the crates. The sow was animated to stand up. When the sow was standing, one person picked up one of her piglets. When the piglet squealed, the dummy arm (plastic hand imitation, Figure 2) was held out to the sow. The sow’s defense and defensive reactions were assessed. The sow’s reaction was classified as being in one of four categories (Table 2).

#### 2.2.6. Trough Cleaning Test (TCT)

In the TCT (2805 observations), the sow’s response to a routine management activity was assessed. The cleaning of the trough was simulated at two defined times. The trough was scraped twice with a feed scoop and the sow’s reaction was recorded. The test was performed at 3 days p.p. (3.4 ± 1.0 days) with closed crates and fixed sows (1444 observations) and at 10 days p.p. (9.8 ± 1.6 days) with loose-housed sows (1361 observations). The sow’s reaction was classified as being in one of three categories (Table 2).

#### 2.2.7. Reunion Test (RT)

On the 3rd day (3.4 ± 1.0 days) p.p., routine procedures were applied to the piglets (iron injection, castration). After that separation, the sow’s reaction (Table 2) upon reunion with her piglets was assessed. Any piglet-related reaction was scored as pleasure to be reunited with the piglets again. This resulted in 1306 observations.

### 2.3. Statistical Analysis

Data were prepared using Microsoft Excel (version 2016, Microsoft Corporation, Redmont, Washington, DC, USA). The statistical analysis of behavioral traits was carried out using Statistical Analysis System SAS 9.4 (SAS Institute Inc., Cary, NC, USA). Means, standard deviations, and the frequency of behavioral traits (e.g., percentage distribution of the categories in the behavioral tests depending on the status of the farrowing pen) were calculated with the procedures MEANS and FREQ. The analysis of variance (ANOVA) and t-test were used to compare the means of behavioral traits between different parity numbers of sows (*n* = 3), the observer performing behavioral tests or observations (*n* = 2), and different statuses of the farrowing crate (closed/open). Due to the small number of sows in higher parities, the sows were divided into three parity classes: 1. class = 1st parity sows; 2. class = 2nd parity sows; 3. class = ≥3rd parity sows. In this experimental setup and the statistical analysis, the effects of crate and point of time could not be separated. However, due to the changes in the point of time of opening the crate, different days of lactation were considered. It could only be assumed that spatial changes within the farrowing pen (with or without crate) may have a higher influence on the behavior of sows than the lactation day. Statistical significance was accepted at *p* < 0.05.

## 3. Results

### 3.1. Nest Building (NB)

In 22.0% of observations (*n* = 803), no nest building activity of sows was documented (category 1, Table 2). Category 2 was recorded in 56.2% (*n* = 2047) of cases. Categories 3 and 4 were observed in 9.1% (*n* = 333) and 12.6% (*n* = 460) of observations, respectively. The NB activity of sows increased significantly in the period from the second (mean ± SD: 1.95 ± 0.82) and first days (2.09 ± 0.80) a.p. to the day of farrowing (2.41 ± 1.04). Significant differences (*p* < 0.05) in the NB activity of sows were found between the two observers (first observer: 2.19 ± 0.93; second observer: 2.05 ± 0.84). There were also significant differences (*p* < 0.05) in the NB activity between the three parity classes of sows (Table 3).

### 3.2. Lying-Down (LDB)

**Figure 3:**
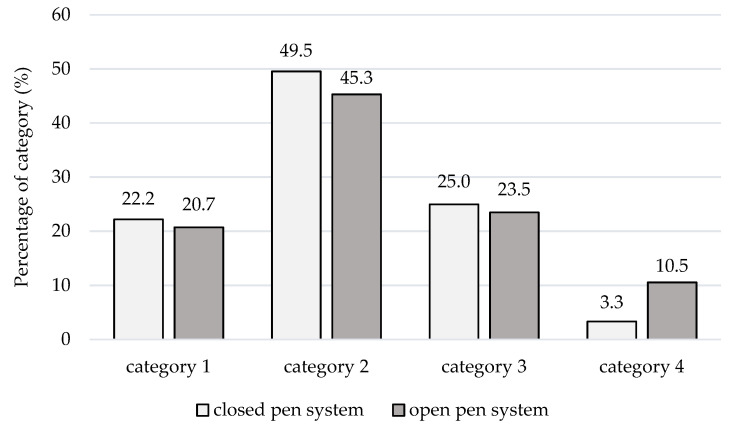
Distribution of the categories in the lying-down behavior (LDB) of gilts (*n* = 1112) depending on the status of the farrowing pen, with categories: 1—very cautious and controlled laying down, 2—cautious laying down, 3—average laying down, 4—careless and fast, uncontrolled laying down.

Category 2 was most frequently documented (49.1%, *n* = 1381 observations), followed by categories 1 (24.3%, *n* = 683), 3 (21.2%, *n* = 596), and 4 (5.5%, *n* = 154) (Table 2). It was observed that the share of category 4 (careless lying-down behavior) increased substantially in the open farrowing crate (8.2%) vs. the closed farrowing crate (2.9%). This trend was especially recognizable in the first parity sows (Figure 3). Significant differences (*p* < 0.05) in the lying-down behavior of sows were found between observers (first observer: 1.99 ± 0.80; second observer: 2.17 ± 0.82) and between different parity classes of animals (Table 3).

### 3.3. Position after Lying Down (PLD)

Category 1 (50.1%, *n* = 709 observations) was most frequently documented. Categories 2 and 3 were recorded in 25.4% (*n* = 360) and 24.5% (*n* = 346) of observations, respectively (Table 2). The status of the crate within the farrowing pen showed no impact on the position of the animals after lying down. Regarding the parity number of sows, the highest value for this trait was found in gilts (Table 3).

### 3.4. Towel Test (TT)

In 42.7% of observations (*n* = 1215), the sows showed a slight reaction to the unexpected situation (Table 1 and Table 2). The share of category 4 (aggressive reaction of sows) increased substantially in the open farrowing crate (5.3 %) vs. the closed farrowing crate (3.7%). The reaction of the sows of parity classes 2 and 3 was more pronounced compared to the reaction of the younger animals (Table 3). The observer had no impact on this trait.

### 3.5. Dummy Arm Test (DAT)

Due to the very low occurrence of category 4 (2.8%) in the data set, categories 3 and 4 were combined to form category 3. Category 1 was most frequently documented (46.2%, *n* = 667 observations), followed by categories 2 (43.1%, *n* = 623) and 3 (10.7%, *n* = 154) (Table 2). Regarding parity classes of sows (Table 3) and the observer performing the test (first observer: 1.67 ± 0.68; second: 1.62 ± 0.65), no significant differences in the reaction of the animals to the stockperson were found.

### 3.6. Trough Cleaning Test (TCT)

**Figure 4:**
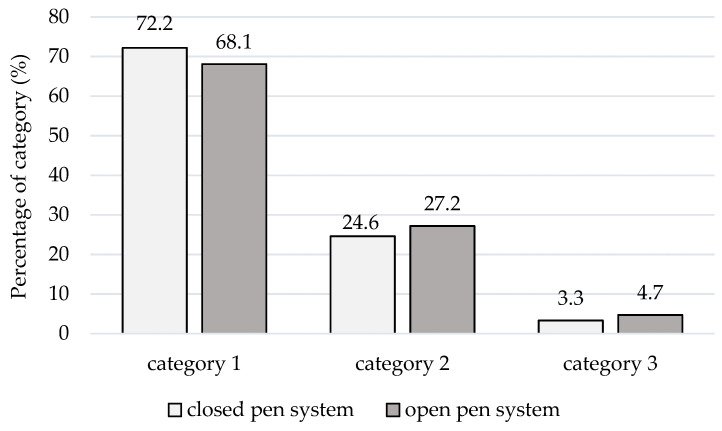
Distribution of the categories in the Trough Cleaning Test (TCT) depending on the status of the farrowing pen, with categories: 1—no reaction to human interaction, 2—little reaction, but without serious background (e.g., sow raises head), 3—strong defensive reaction (e.g., tries to bite stockperson).

In 70.2% of observations (*n* = 1969), category 1 was documented (Table 1 and Table 2). The share of category 3 (strong defensive reaction of sows) increased in the open vs. closed farrowing crate (Figure 4). Significant differences (*p* < 0.05) in the reaction of sows to stockpersons during trough cleaning were identified between the observers performing TCT (first observer: 1.22 ± 0.50; second observer: 1.45 ± 0.57) and between different parity classes of animals (Table 3).

### 3.7. Reunion Test (RT)

In 61.8% of cases (*n* = 807), the sows reacted with joy upon the reunion with their piglets after a treatment. No reaction of sows was recorded in 38.2% (*n* = 499) of observations (Table 1 and Table 2). The sows of the first and second parity classes showed more joy in being reunited with their piglets than older animals (Table 3).

## 4. Discussion

The demands on lactating sows in husbandry systems without fixation are particularly high as they should show maternal behavior, but not be too aggressive, to enable easy handling. On the one hand, the sow should be calm and gentle enough to present no risk to the personnel; on the other hand, she should be protective and careful towards her piglets. To consider these traits in a selection process for breeding, or to simply check whether the sows are suitable for a system without long-term fixation, standardized observations and tests can be of great benefit. In this study, such observational procedures and tests were tested and evaluated.

Concerning nest building (NB) activity, the domestication of sows led to no changes in the motivation of the animals to show nest building behavior close to birth [19]. While the activity itself is not essential to assessing the sow’s suitability for loose-housing systems, the degree of nest building behavior can hint at her willingness to accept provided material and her motivation to perform such behavior. It was shown that sows with pronounced nest building behavior had less complication at parturition, and fewer stillborn piglets in the litter [13]. No reduction in nest building behavior was observed when sows were offered a pre-constructed nest, which indicates that the building activity itself is a behavioral need of the prepartal sow [20]. In the present study, most prepartal sows showed a high interest in the offered nesting material (gunnysacks). Without appropriate building material, sows try to perform nest building behavior using crate equipment, leading to frustration and even decreased reproductive performance [13]. If enough material is offered, increased motivation and nest building activity is reported [21]. Nest building behavior begins within the last 30 to 24 h before farrowing and is conducted more intensively in the last 12 to 6 h before birth [22,23]. Sows usually show the highest activity in the 24 h before farrowing [24,25]. This trend was observed in the present study as well, with activity increasing as the birth approached. Between the assessments of the two observers, a significant difference was found; this can often be observed in behavioral tests. Moreover, the parity class influenced the reaction of the sow, with sows in the second parity showing slightly increased nest building activity.

Regarding lying-down behavior (LDB) and position after lying down (PLD), most sows showed cautious lying behaviors. Most studies report that the majority of piglet loss is related to position changes of the sows from standing to lying, including rolling [6,26]. These position changes are prevented by farrowing crates, which slow down uncontrolled lying down movements and thereby reduce the risk of crushing piglets [27]. This control by the crate is reflected in the present study, with an increase in category 4 LDB in open farrowing pens. A similar increase in activity levels of loosed house sows was also reported in another study [28]. In systems without permanent fixation, the LDB is an important behavioral trait of sows. It was shown that it is beneficial if sows can carefully perform a series of pre-lying behaviors to warn the piglets before they actually lie down, rather than fall straight down [18]. In terms of the number of changes from standing to lying, the frequency of changes was not related to the occurrence of dangerous events or crushing [29]. With regard to the position of the sow after lying down (PLD), the stomach-teats position was the preferred position, and even more so with higher parities. Similar results were found in another study investigating the method of lying down [30].

Concerning the tests related to human-animal interactions and new situations, the results of the present study indicate that in the examined population, the sows were calm and tolerant of human contact. For handling in routine situations, sows with low activity, which are neither too aggressive nor too passive, are advantageous [31]. It was reported that sows with less fear of humans had higher reproductive success and more adaptive maternal behavior [32]. All designed tests (TT, DAT, TCT) were suitable for characterizing the sows’ behavior towards novel objects, human interaction, and unknown situations. The results of TT and DAT did not differ between the two observers. The sows showed mostly no to only slight defensive reactions towards the suddenly appearing towel (TT), the dummy arm test (DAT), and the cleaning of the trough (TCT). The numbers of attacking sows were very low. In this study, DAT was performed in closed crates only. This point of time was chosen to keep possible impacts on piglets to a minimum. Therefore, the test was performed when routine procedures in piglets were conducted. In further studies, other points in time could be examined.

Sows in loose-housing systems were shown to react more strongly to human interaction than did animals in crates [33]. Similar results were assessed in the present study, with stronger reactions in TT and TCT after the crates were opened. The animals reacted with a slightly increased defensive behavior. In the present results, an increase in the mean values of behavior to unknown situations during a routine procedure was observed with an increase in the parity class. This is in agreement with Marchant Forde [33], documenting an increase in aggressive behavior with higher parity classes. One cause of this could be that high-parity sows react more mistrustfully to human handling measures due to more negative experiences (e.g., vaccination), as well as the fact that an older sow has a more fully developed personality, represented, e.g., by a higher position in the hierarchy of a group.

In the present study, the sows with a higher parity class reacted in DAT slightly more strongly to the simulated piglet treatment and the induced piglet scream, even if no significant differences were found between the litter classes. In other studies, sows with higher parities were associated with a stronger withdrawal response to humans as well [34] and were more fearful of humans and crushed more piglets [11]. In other studies, it was primarily the gilts that reacted more strongly to the screaming of piglets [10,16,35].

The Reunion Test (RT) was performed to characterize the maternal behavior, which includes the response to piglet signals and defending them against enemies, which is also an important maternal characteristic. In previous studies, sows were observed when they were separated from their piglets and when they were reunited [12,16,36]. Their maternal behavior was characterized in situations in which their piglets were treated or stimulated to scream [4,10,16]. In a study by Løvendahl et al. [15], most sows showed a distinct reaction combined with vocalization—but no serious attacks—if piglets were treated. In the present study, the gilts showed the highest mean values during the reunion in RT with their piglets, similar to the results for gilts in a separation test in a previous study [16]. A potential reason for this could be that inexperienced gilts were not familiar with the routine treatments soon after farrowing and therefore reacted more strongly to the reunion.

## 5. Conclusions

The results of this study suggest that the behavioral observations and developed behavioral tests are suitable for assessing nesting activity before birth, determining lying down behavior, and recording behavior towards humans and piglets, routine measures, and unknown situations. The assessed traits are essential to characterize sows’ behavior in loose-housing systems and their suitability concerning the respective challenges of these systems. During the behavioral tests, most of the sows showed calm and kind reactions to human interactions. The number of attacking sows was very low, but not zero. All observations and tests are easy to conduct in practice and provide useful information. Due to their good practicability, these tests are beneficial for selecting less fearful, gentle sows to increase the welfare of sows and their piglets in loose-housing systems, even so for the stockperson. Further investigations will include an analysis of the genetic aspects of the behavioral traits of lactating sows.

## Figures and Tables

**Table 1:** Number of performed behavioral observations and tests, and number of phenotyped sows (N).

Behavioral Tests and Observations	Number of Tests and Observations	
	Closed Farrowing Crate	Open Farrowing Crate
Nest building (NB)	3643 N = 770	-
Lying-down behavior (LDB)	1444 N = 770	1370 N = 747
Position after lying down (PLD)	701 N = 464	714 N = 471
Towel Test (TT)	1444 N = 771	1402 N = 756
Dummy Arm Test (DAT)	1444 N = 770	-
Trough Cleaning Test (TCT)	1444 N = 771	1361 N = 742
Reunion Test (RT)	1306 N = 713	-

**Table 2:** Description of behavioral observation procedures and behavioral tests with sows’ reaction categories.

Behavioral Observation/Test	Behavioral Traits and Time of Evaluation	Description of Categories
Nest building (NB)	Manipulation of the gunnysack 2 d a.p., 1 d a.p., 0 d (day of farrowing)	1—no reaction, ignoring the sack, removing the sack out of the sow’s area 2—hanging gunnysack chewed through and manipulated 3—sow lies on gunnysack 4—gunnysack in trough 5—gunnysack was torn
Lying-down behavior (LDB)	Manner of laying down behavior 3 d p.p., 19 d p.p.	1—very cautious and controlled laying down 2—cautious laying down 3—average laying down 4—careless and fast, uncontrolled laying down
Position after lying down (PLB)	Favorite position after lying down 3 d p.p., 19 d p.p.	1—on stomach and teats 2—on the right body side 3—on the left body side
Towel Test (TT)	Sow’s reaction to novel object and unexpected situation 3 d p.p., 10 d p.p.	1—no object related reaction (e.g., sow remains in lying position) 2—slight reaction (e.g., raises her head, but remains lying) 3—medium reaction (e.g., sow sits up) 4—strong defensive reaction, (e.g., sow stands up, is nervous and aggressive)
Dummy Arm Test (DAT)	Sow’s reaction to stockpersons when piglets were handled 4 d p.p.	1—no object related reaction (e.g., sow looks maximally after her piglet) 2—slight reaction, without serious background (e.g., sow raises head) 3—strong reaction (sow threatens, defensive reaction without bite) 4—very strong object-related reaction (sow bites the dummy arm)
Trough Cleaning Test (TCT)	Sow’s reaction to stockpersons during trough cleaning 3 d p.p., 10 d p.p.	1—no reaction to human interaction 2—little reaction, but without serious background (e.g., sow raises head) 3—strong defensive reaction (e.g., tries to bite stockperson)
Reunion Test (RT)	Sow’s reaction to a reunion with piglets 3 d p.p.	1—no reaction 2—with reaction (e.g., looking for piglets, grunting)

d a.p. = days ante partum; d p.p. = days post partum.

**Table 3:** Reaction of the sows during behavioral observations and tests depending on the parity number (mean ± SD) with *n* = number of observations.

Behavioral Observations and Tests	Parity Classes of Sows		
	1st Parity	2nd Parity	≥3rd Parity
Nest building (NB)	2.11 ± 0.87 *n* = 1545	2.20 ^a^ ± 0.88 *n* = 981	2.07 ^b^ ± 0.94 *n* = 1117
Lying-down behavior (LDB)	2.16 ^a^ ± 0.84 *n* = 1112	2.05 ^a^ ± 0.79 *n* = 782	2.01 ^b^ ± 0.81 *n* = 920
Position after lying down (PLD)	1.82 ^a^ ± 0.82 *n* = 591	1.73 ± 0.82 *n* = 395	1.66 ^b^ ± 0.82 *n* = 429
Towel Test (TT)	2.06 ^a^ ± 0.83 *n* = 1115	2.19 ^b^ ± 0.80 *n* = 790	2.24 ^b^ ± 0.84 *n* = 941
Dummy Arm Test (DAT)	1.63 ± 0.63 *n* = 570	1.65 ± 0.67 *n* = 400	1.65 ± 0.70 *n* = 474
Trough Cleaning Test (TCT)	1.26 ^a^ ± 0.48 *n* = 1098	1.33 ^b^ ± 0.53 *n* = 782	1.44 ^c^ ± 0.63 *n* = 925
Reunion Test (RT)	1.69 ^a^ ± 0.46 *n* = 522	1.64 ^a^ ± 0.48 *n* = 365	1.51 ^b^ ± 0.50 *n* = 419

^a-b-c^ Values within row with different superscripts differ significantly (*p* < 0.05).

## Data Availability

The data presented in this study are available on request from the corresponding author. The data are not publicly available due to privacy reasons related to BHZP GmbH.
